# Hair Growth-Promoting Effect of a Horse Oil–Folium Isatidis Topical Preparation via Modulation of VEGF, TGF-β1, and β-Catenin Signaling in C57BL/6 Alopecia Mice: A Pilot Study

**DOI:** 10.3390/cimb48090960

**Published:** 2026-09-19

**Authors:** Miaomiao Qiao, Jie Luo, Shijie Bi, Peng Gao, Jinfang Zhu, Juan Wang

**Affiliations:** 1 College of Food Science and Pharmacy, Xinjiang Agricultural University, Urumqi 830052, China; qiaoyx2026@126.com (M.Q.); 13525016196@163.com (J.L.); bsj19941001@163.com (S.B.); gaop2026@126.com (P.G.); 2 Xinjiang Uygur Autonomous Region Special Fruits and Vegetables Processing Engineering Research Center, Urumqi 830052, China

**Keywords:** alopecia, horse oil, folium isatidis, growth-promoting

## Abstract

Wnt/β-catenin signaling is a key regulator of hair follicle cycle progression, and aberrant activation or suppression of this pathway has been implicated in the pathogenesis of alopecia. Horse oil (HO) and Folium Isatidis (FI) have been traditionally used in East Asian medicine for hair growth, but their combined molecular mechanism remains unclear. We evaluated a topical preparation combining HO and FI (HOFI) in two C57BL/6 mouse models: physiological depilation (telogen-to-anagen transition) and testosterone-induced seborrheic alopecia. Hair growth was assessed by gross appearance, hair length, and histomorphometry (follicle number, size, dermal thickness). Plasma and skin levels of vascular endothelial growth factor (VEGF) and transforming growth factor-β1 (TGF-β1) were measured by ELISA. Protein expression of β-catenin, Bcl-2, and Bax in skin tissue was analyzed by Western blotting. Compared with NC, HO, FI, and HOFI significantly increased hair length, follicle density and size, and dermal thickness (*p* < 0.05). All three treatments upregulated VEGF and β-catenin, suppressed TGF-β1, elevated Bcl-2, and reduced Bax expression. HOFI exhibited significantly greater effects than either monotherapy across all endpoints (*p* < 0.05). HOFI promotes hair growth by activating the VEGF/β-catenin signaling axis, inhibiting TGF-β1, and shifting the Bcl-2/Bax balance toward follicular cell survival. These findings provide a molecular rationale for developing HO–FI combinations as topical agents for alopecia.

## 1. Introduction

Hair loss is a common and distressing condition affecting a large proportion of middle-aged adults globally [1]. Appearance is a means of expressing confidence, and hair plays an important role superficially as the first part of the body seen by others [2]. Hair loss refers to the phenomenon of reduced hair density or thinning of hair strands, which may be induced by aesthetic and psychological factors, including stress, aging, and imbalances in hormones and nutrients. Although hair loss is not life-threatening, it affects self-esteem and quality of life [3]. There are two main types of hair loss: physiological and seborrheic alopecia. Physiological alopecia is an autoimmune inflammatory dermatological condition characterized by non-scarring hair loss on the scalp. Physiological alopecia is caused by problems with the inflammatory process, oxidative stress, cognitive variables, infections, comorbidities, and microecological imbalance [4]. Seborrheic alopecia is a common non-scarring hair loss condition that causes hair to weaken and shrink. Also known as male pattern baldness, this condition is characterized by progressive hair follicle miniaturization [5]. Seborrheic alopecia is influenced by a variety of factors, such as genetics, age, sleep, nutrition, sun exposure, and scalp health [6]. The main approaches to alopecia treatment are largely classified into two main categories: androgen and non-androgen medicines. Pharmacological options targeting androgen signaling comprise 5α-reductase inhibitors (e.g., finasteride, dutasteride) and anti-androgen agents such as alfatradiol [7]. The main non-androgen medicine for alopecia treatment is minoxidil [8]. However, various side effects will occur (including scalp irritation, itching, dryness, excessive facial, body hair growth and sexual dysfunction) if androgen and non-androgen medicines for alopecia treatment are used long-term [5,9]. There are inherent advantages of natural products, including their affordability, convenience, and minimal toxic and adverse effects, which have received attention from researchers. Herbal remedies for alopecia have a profound historical foundation during different periods of civilizations, including Ancient Egypt, Traditional Chinese Medicine, Ayurveda, and medieval European traditions. Black seed oil and rosemary oil were the traditional herbal medications that were employed to enhance hair vitality, scalp health, and hair growth [10]. The extension of the anagen phase following topical caffeine application is attributed to its antagonistic effect on targeted receptors [11]. Tea is rich in epigallocatechin and gallate, which possess potent antioxidant and anti-inflammatory properties, and promotes hair growth by inhibiting dihydrotestosterone production and directly stimulating hair follicle cells [12]. Curcumin may promote hair growth by regulating the hair follicle growth cycle [13]. Ginsenosides Rb1 and Rg3 in ginseng have been found to activate the β-catenin pathway, thereby enhancing hair growth [14,15]. Horse oil (HO) is a kind of subcutaneous oil extracted from horses [16,17] which is rich acids, including linoleic acid, palmitoleic acid, arachidonic acid, etc. [16,18,19]. Linoleic acid, a metabolic precursor of arachidonic acid, promotes proliferation of human dermal papilla cells through β-catenin pathway activation [20]. Furthermore, arachidonic acid can increase proliferation of human dermal papilla cells and promote hair growth in a seborrheic alopecia mouse model [21]. Folium isatidis (FI) is the leaf of *Isatis tinctoria* L. [22], in which there are mainly active ingredients such as indigotin and indirubin. Indirubin, one of the major active constituents, promotes hair growth by regulating the β-catenin signaling pathway [23]. In this study, the hair growth-promoting effects of horse oil and folium isatidis eyebrow pencil (HOFI) were investigated in mouse models of physiological and seborrheic alopecia by evaluating hair length, dermal thickness, and histopathology. Using commercial ELISA kits, we quantified the concentrations of vascular endothelial growth factor (VEGF) and transforming growth factor-β1 (TGF-β1) in both plasma and skin tissue. Meanwhile, the expression levels of β-catenin, Bcl-2, and Bax proteins in skin tissue were analyzed using Western blot to explore the potential mechanisms. In the future, HOFI will be a prospective natural compound preparation to prevent and treat hair loss.

## 2. Materials and Methods

### 2.1. Chemicals, Materials, and Animals

Testosterone propionate was obtained from the Hangzhou Animal Pharmaceutical Factory (Hangzhou, China). Electrophoresis buffer, electroporation buffer (Servicebio), and markers were purchased from Beijing Botaisi Biology (Beijing, China). β-catenin rabbit anti-monoclonal antibody, Bcl-2 rabbit anti-monoclonal antibody, Bax rabbit anti-monoclonal antibody, and internal reference GAPDH were purchased from Cell Signaling Technology Corporation (Danvers, MA, USA). IRDye 800CW goat anti-rabbit IgG secondary antibody was purchased from LI-COR Biosciences (Lincoln, NE, USA). TGF-β1 and VEGF ELISA kits were purchased from Nanjing Jiancheng Bioengineering Institute (Nanjing, China). Soybean oil was purchased from Zhejiang Tianyu Yam Oil Co., Ltd. (Zhejiang, China). Osman’s eyebrow pencil was purchased from Osman Ltd. (Urumqi, China). Minoxidil solution was purchased from Zhejiang Wansheng Pharmaceutical Co., Ltd. (Zhejiang, China). Heparin sodium centrifuge tubes were obtained from Yangpu Medical Technology Co., Ltd. (Guangzhou, China). 4% paraformaldehyde was purchased from Beyotime Biotechnology (Shanghai, China). BCA Protein Quantification Kit was obtained from Kangwei Century Co., Ltd. (Jiangsu, China). High-efficiency RIPA lysis buffer, 4× protein loading buffer, TBST buffer, and Western blot blocking solution were obtained from Beijing Solarbio Science & Technology Co., Ltd. (Beijing, China). SDS-PAGE Gel Rapid Preparation Kit was purchased from Wuhan Servicebio Technology Co., Ltd. (Wuhan, China). AR-grade chemicals were procured for the proposed analysis. Hair removal cream was purchased from Veet (Paris, France). All other reagents used were of analytical grade and commercially available.

Healthy C57BL/6 mice (20 ± 2 g) were provided by the Experimental Animal Center of Xinjiang Medical University. After the experiment, animals were anesthetized and killed by cervical dislocation, and hair as well as dorsal skin tissues were collected. The study protocols were approved by the Animal Welfare and Ethics Committee of Xinjiang Agricultural University (No. 2024-0001) and performed in line with the International Guide for the Care and Use of Laboratory Animals.

### 2.2. Preparation of HO

Subcutaneous fat from 2-year-old local meat-type horses (*Equus caballus*) in Ili Prefecture, China, was collected within 30 min post-slaughter. No horses were euthanized specifically for this study; subcutaneous fat was obtained as a by-product of routine meat production. Minced fat (1:1 *w/v* with water) was enzymatically hydrolyzed with neutral protease (2%, *w*/*w*) at 40 °C for 2 h, incorporating tea polyphenols (0.3 g/kg fat), citric acid (0.4 g/kg fat), water soluble rosemary extract (0.12 g/kg fat), and rosemary essential oil (0.03 g/kg fat). The emulsion was centrifuged (4500 r/min, 15 min), the supernatant collected, stabilized with ascorbyl tetraisopalmitate (0.08 g/kg oil), and stored at −20 °C.

### 2.3. Preparation of FI Extract

Fresh leaves of *Isatis indigotica* Fort. (Huocheng County, Ili, China; authenticated by Prof. Jinfang Zhu, Xinjiang Agricultural University) were rinsed, surface-dried, and juiced using a masticating juicer without added water. The expressed juice was filtered through four layers of medical gauze, concentrated under reduced pressure at 60 °C to a thick paste, and stored at 4 °C until use.

### 2.4. Preparation of HOFI

The HOFI formulation contained 10% (*w*/*w*) HO and 18% (*w*/*w*) FI (HO:FI = 5:9, *w*/*w*) in a wax-based vehicle. To prepare the eyebrow pencil, FI extract was mixed with diisostearyl malate, pentaerythrityl tetraisostearate, and Tween 80, then heated at 80 °C. Separately, HO, lecithin, paraffin wax, candelilla wax, carnauba wax, Fe_3_O_4_, and Fe_2_O_3_ were melted at 80 °C. The two portions were mixed, poured into molds, and cooled at −20 °C for 30 min. Indigo and indirubin in the final product (batch 2024.07.06c) were 1137.82 ± 37.18 μg/g and 79.56 ± 0.92 μg/g, respectively (HPLC).

### 2.5. Chemical Characterization

Indigo and indirubin in the final HOFI eyebrow pencil were qualitatively identified by TLC and quantitatively analyzed by HPLC-PDA. Chromatographic separation was performed on an InertSustain C18 column (150 mm × 4.6 mm, 5 μm) with methanol–water (75:25, *v*/*v*) at 1.0 mL/min, 30 °C, 20 μL injection, and detection at 289 nm.

### 2.6. Experimental Method

#### 2.6.1. Establishment of a Mouse Model of Physiological Alopecia

**Figure 1:**
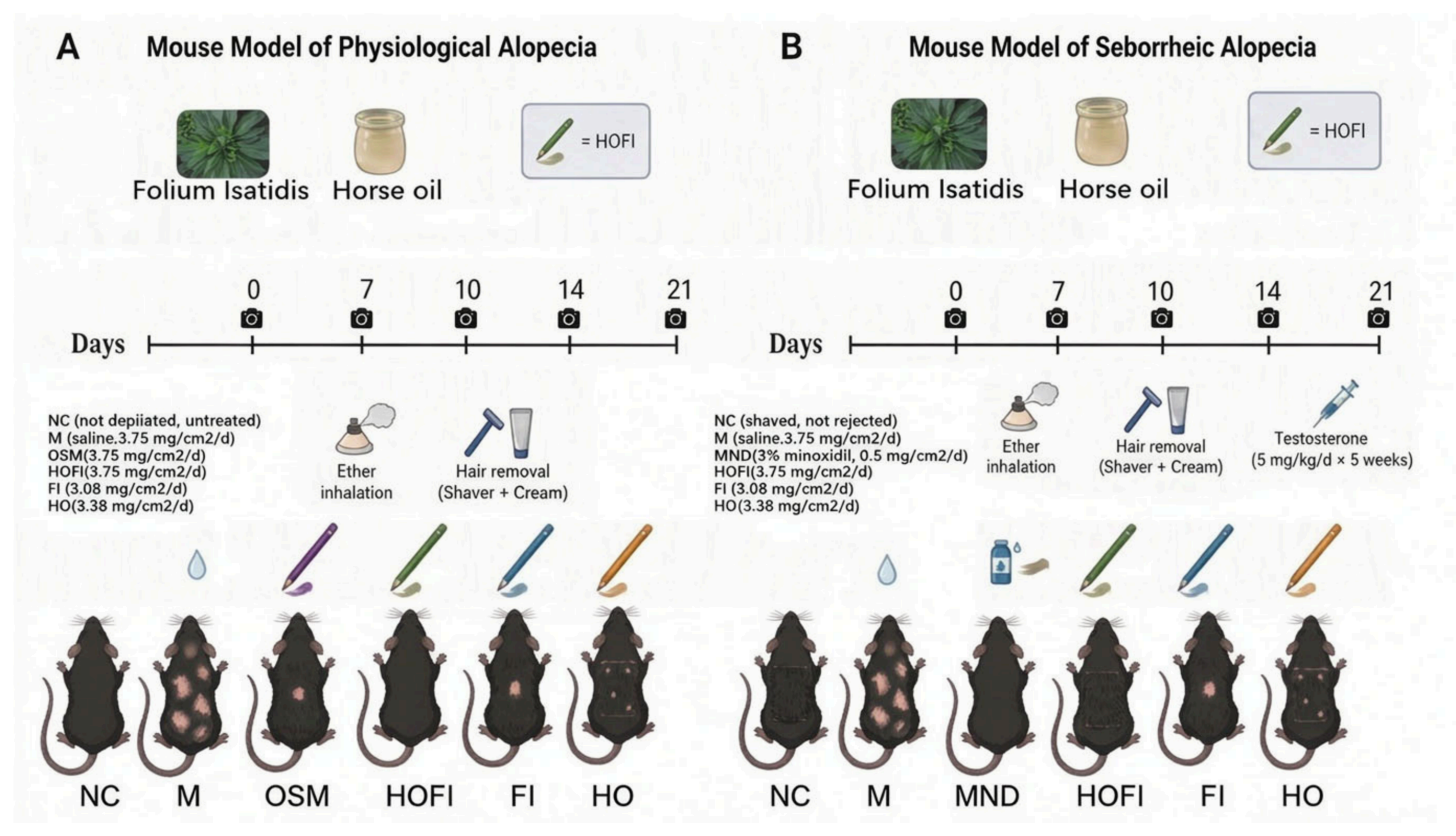
Experimental design for the physiological and seborrheic alopecia mouse models.

Figure 1 provides a schematic overview of the animal experimental design. Animals were assigned randomly to six cohorts (*n* = 12 each): normal control (NC), model (M), Osman’s eyebrow pencil (OSM), horse oil–Folium Isatidis eyebrow pencil (HOFI), Folium Isatidis eyebrow pencil (FI), and horse oil eyebrow pencil (HO).

All mice, except those in the NC group, were anesthetized by ether inhalation. A 4 × 2 cm^2^ dorsal site was trimmed using a hair clipper, followed by application of a depilatory cream to eliminate remaining hairs. The M group, the OSM group, the HOFI group, the FI group, and the HO group were smeared with the product on the depilatory areas every day. Photographs were taken regularly to record hair growth changes on the 0, 7th, 14th, and 21st days.

#### 2.6.2. Administration Methodology for a Mouse Model of Physiological Alopecia

Seventy-two mice were randomly divided into six groups: NC group (no depilation, no treatment, normal control), M group (saline, 3.75 mg/cm^2^/d, vehicle control for normal hair regrowth after depilation), HO group (3.38 mg/cm^2^/d), FI group (3.08 mg/cm^2^/d), HOFI group (3.75 mg/cm^2^/d), and OSM group (3.75 mg/cm^2^/d).

#### 2.6.3. Establishment of a Mouse Model of Seborrheic Alopecia

The mice were randomly divided into six groups (n = 12): the normal control group (NC group), the Model group (M group), the Positive Control (Minoxidil) group (MND group), the Horse Oil Folium Isatidis eyebrow pencil group (HOFI group), the Folium Isatidis eyebrow pencil group (FI group), and the Horse Oil eyebrow pencil group (HO group). All mice were anesthetized with ether, and an area of approximately 4 × 2 cm^2^ on the dorsal skin was shaved using a hair shaver, followed by removal of residual hair with a depilatory cream. Mice in all groups except the NC group received daily subcutaneous injections of testosterone (5 mg/kg/d) for 5 weeks to establish the seborrheic hair loss model. Subsequently, the dorsal skin of mice in the respective groups was topically treated once daily for 21 consecutive days as follows: the M group received saline; the positive control group received 3% MXD; the HOFI, FI, and HO groups received their respective formulations. Photographs were taken regularly to record hair growth changes on the 0, 7th, 14th, and 21st days.

#### 2.6.4. Administration Methodology for a Mouse Model of Seborrheic Alopecia

Seventy-two mice were randomly divided into six groups: NC group, M group (saline, 3.75 mg/cm^2^/d), HO group (3.38 mg/cm^2^/d), FI group (3.08 mg/cm^2^/d), HOFI group (3.75 mg/cm^2^/d), and MND group (3% minoxidil, 0.5 mg/cm^2^/d).

#### 2.6.5. Hair Growth Measurement

The mice were selected to evaluate the mouse hair color in the depilated dorsal region on the 0, 7th, 10th, 14th, and 21st days. Meanwhile, hair length in the area of hair removal was measured on the 0, 7th, 14th, and 21st days. At the last administration, feeding stopped in all groups for approximately 12 h, and then the blood was collected into heparinised centrifugal tubes by removing the eyeball. Blood samples were centrifuged at 390× *g* for 10 min to isolate plasma, which was subsequently stored at −80 °C. Skin tissue was stripped for histopathological examination and analysis of VEGF, TGF-β1, β-Catenin, Bcl-2 and Bax expression.

#### 2.6.6. Histological Analysis

The skin tissue was fixed in 4% formaldehyde overnight, embedded and prepared into serial sections (4 μm thick). The paraffin sections of skin tissue were stained by hematoxylin–eosin (HE) and observed for the morphological alternations by microscope.

#### 2.6.7. ELISA Detection of VEGF and TGF-β1 Levels in Plasma and Skin Tissue

Homogenates of skin tissue were processed by centrifugation at 14,010× *g* for 5 min to obtain the supernatant. ELISA was then performed to quantify VEGF and TGF-β1 in both skin and plasma samples according to kit-provided guidelines.

#### 2.6.8. Western Blot Analysis of β-Catenin, Bcl-2 and Bax Expression Levels

Skin tissues were diced and lysed with a RIPA buffer kit to isolate total protein. The lysates were centrifuged at 14,010× *g* for 5 min at 4 °C. Protein concentration in the supernatant was determined using a BCA assay kit, normalized to a uniform concentration, and denatured by heating in boiling water for 5 min. Equal protein loads were resolved by 10% SDS-PAGE and electroblotted onto polyvinylidene fluoride (PVDF) membranes. The PVDF membrane of skin tissues was incubated with specific primary antibody β-Catenin (1:1500), Bcl-2 (1:1500), Bax (1:1500), and GAPDH (1:1000) overnight at 4 °C. Then, the membranes were incubated with IRDye 800CW Goat anti-Rabbit (1:20,000) for 1 h at 37 °C. The primary antibody GAPDH served as a loading control and the target band optical density was detected using a two-color infrared imaging system. The data were quantified using Image J 8.0 software.

### 2.7. Statistical Analysis

Results are shown as the mean ± SD. Statistical evaluations were conducted using GraphPad Prism 8. Group differences in pathological sections, dermal thickness, VEGF, TGF-β1, β-Catenin, Bcl-2, and Bax were assessed via one-way ANOVA, with Tukey’s post hoc test applied afterward. Hair length data were evaluated using two-way ANOVA followed by the Bonferroni post hoc test. A *p*-value below 0.05 or 0.01 indicated statistical significance.

## 3. Results

### 3.1. Chemical Characterization of HOFI

**Figure 2:**
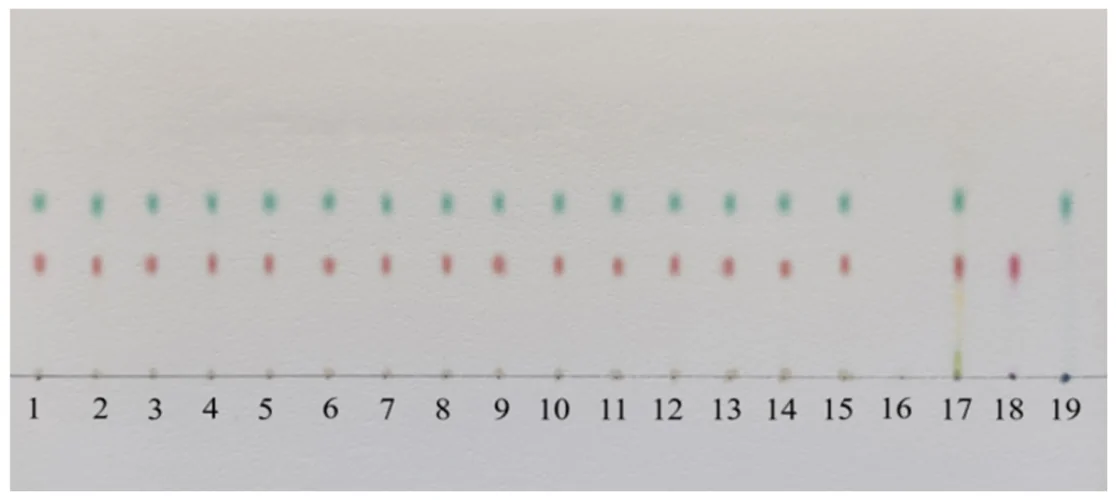
TLC chromatogram of HOFI. Lanes 1–15: test samples; lane 16: negative control; lane 17: *Isatis indigotica* Fort. reference herbal material; lane 18: indirubin reference standard; lane 19: indigo reference standard.

The TLC identification results of HOFI are shown in Figure 2. In the chromato-gram of the test sample, spots of the same color appeared at positions corresponding to the indigo and indirubin reference standards and the *Isatis indigotica* Fort. (Folium Isatidis) reference herbal material, while the negative control showed no interference.

**Figure 3:**
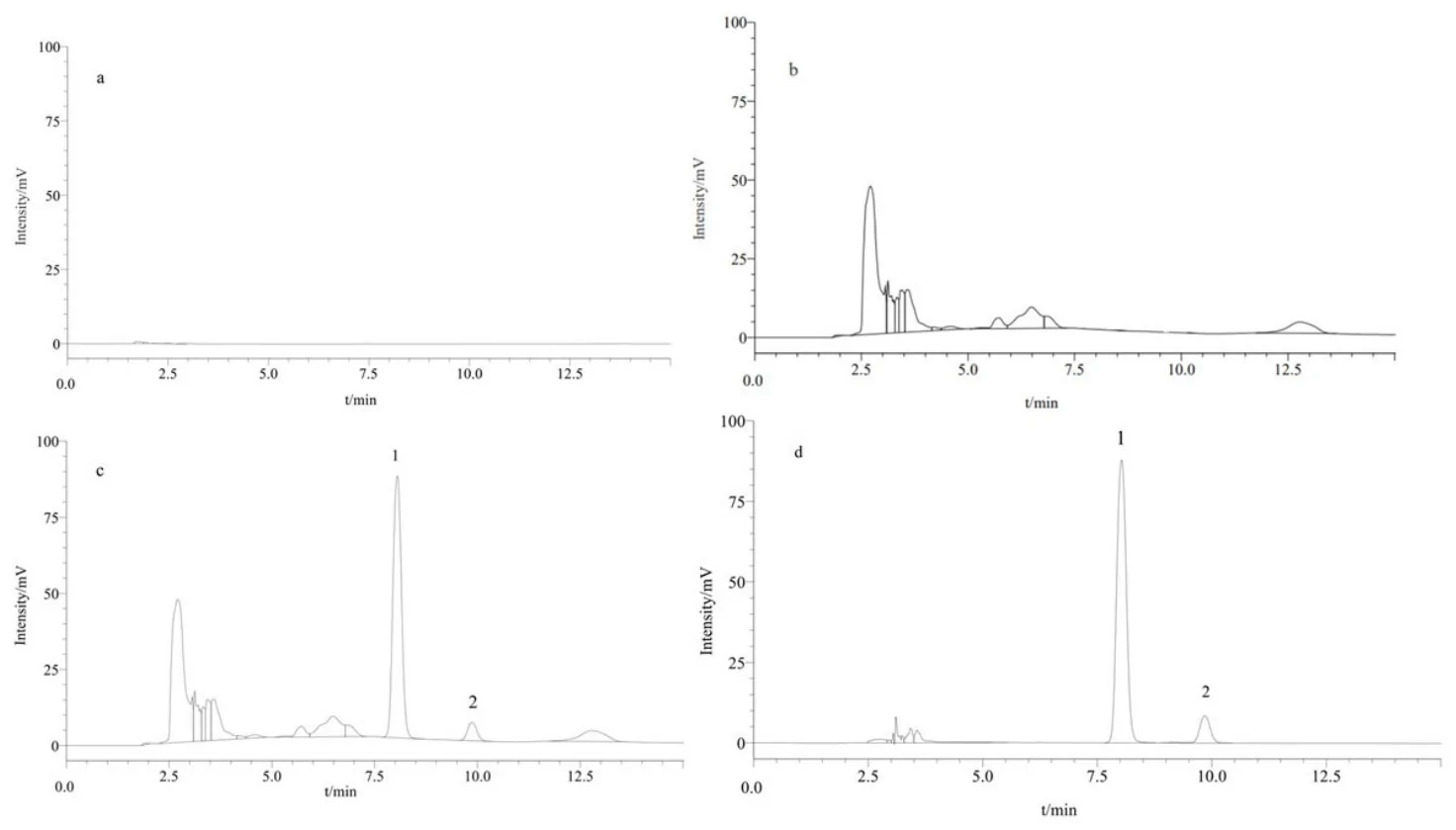
HPLC-PDA chromatograms of indigo and indirubin. (**a**) Blank solvent; (**b**) negative control; (**c**) test sample solution; (**d**) mixed reference standards. Peak 1: indigo; peak 2: indirubin.

The HPLC results showed that the chromatographic peaks eluted rapidly with good symmetry, with a resolution > 1.5 and theoretical plate number > 5000. Neither the blank solvent nor the negative control produced interfering peaks, confirming that the chromatographic conditions met the requirements (Figure 3).

Accurately weighed samples from 15 batches were prepared as test sample solutions according to the described procedure, injected into the HPLC system, and the peak areas were recorded to calculate the content of each batch. The results showed that the indigo content in the 15 batches ranged from 1104.973 to 1194.580 µg/g, and the indirubin content ranged from 73.540 to 81.628 µg/g. The results are summarized in Table 1.

### 3.2. Effects of HOFI on Promoting Hair Growth in Physiological Alopecia Mice

#### 3.2.1. Effects of HOFI on Hair Growth Situations in Physiological Alopecia Mice

**Figure 4:**
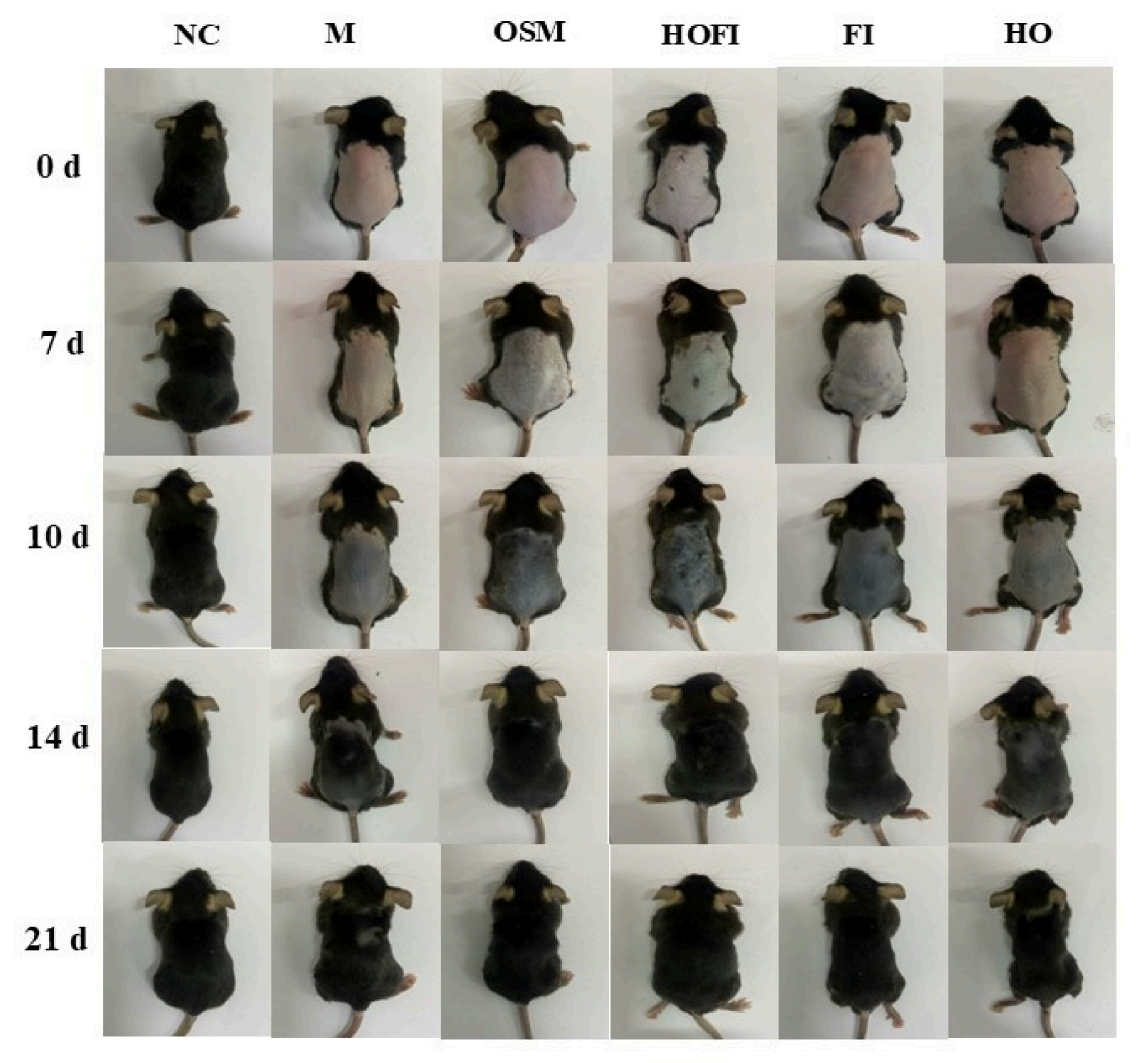
Effects of HOFI on hair growth situations in physiological hair loss mice.

Artificial depilation was performed on the dorsal skin of 7–8-week-old C57BL/6 mice. At this age, follicles are synchronized in the telogen stage; depilation mechanically removes telogen hairs and induces a synchronous anagen phase [24]. The non-depilated control group remained minimally pigmented throughout the 12-day study, confirming baseline telogen status, whereas all depilated groups exhibited progressive skin darkening indicative of anagen induction [25]. During the hair cycle, the color in the depilated dorsal region gradually shifted from pink to dark because of hair growth [26]. As shown in Figure 4, the hair in the dorsal region of the NC group was not depilated, and exhibited black and dense hair. Meanwhile, the pink skin was observed in the depilated dorsal region of the mice in other five groups, indicating that their hair cycle had entered the telogen phase. The above results showed that the mouse model of physiological alopecia was successfully established. On day 7 post-depilation, the depilated dorsal skin of the M group appeared light gray, indicating sparse hair regrowth at the normal physiological rate. In comparison, the HO, FI, OSM, and HOFI groups exhibited progressively darker skin coloration, reflecting accelerated anagen progression and denser hair regrowth relative to the vehicle control baseline. After interventions from the 7th to the 21st day, the depilated dorsal region of the mice in all groups displayed gradually darker colors, indicating that more hair had grown. Meanwhile, the depilated dorsal region of the mice in the OSM, HOFI, FI, and HO groups displayed darker colors than those in the M group, indicating that there is more hair in the OSM, HOFI, FI, and HO groups than in the M group. Furthermore, the depilated dorsal region of the mice in the HOFI group displayed darker colors than those in the OSM, FI, and HO groups, indicating that HOFI could promote a faster transition of the hair cycle from the telogen phase to the anagen phase. Additionally, on the 7th day, the hair of the mice in each group had not yet grown, making it impossible to measure hair length. Based on the results of the preliminary experiment, 10 days was chosen as the starting point for measuring hair length.

#### 3.2.2. Effects of HOFI on Hair Length in Physiological Alopecia Mice

As shown in Table 2, on the 10th, 14th and 21st days of intervention, the hair length of mice in the M group was significantly shorter than that in the NC group (*p* < 0.01). These results confirmed that depilation successfully induced a synchronized anagen phase, establishing the desired model for evaluating hair growth promotion. On the 10th day of intervention, the hair length in the HO group was not significantly different from that in the M group (*p* > 0.01). On the 14th and 21st days of intervention, the hair length of mice in the HO group was longer than that in the M group (*p* < 0.01). The hair length of mice in the OSM, HOFI, and FI groups was significantly longer than that in the M group (*p* < 0.01). Furthermore, the hair length of mice in the HOFI group was significantly longer than that in the OSM group (*p* < 0.01). Notably, the hair length of mice in the HOFI group was significantly longer than that in the FI or HO groups (*p* < 0.01). Collectively, these results demonstrate that HOFI effectively promotes hair growth. Notably, its efficacy was superior to that of the OSM, as well as to the individual treatments with HO or FI alone. Significant hair growth-promoting effects were observed in the OSM, HOFI, and FI groups after 10 days of intervention. However, the hair growth-promoting effect only provided evidence in the HO group after 14 days of intervention, indicating a slower onset of action in the HO group.

#### 3.2.3. Regulation Effects of HOFI on Hair Follicles in Physiological Alopecia Mice

**Figure 5:**
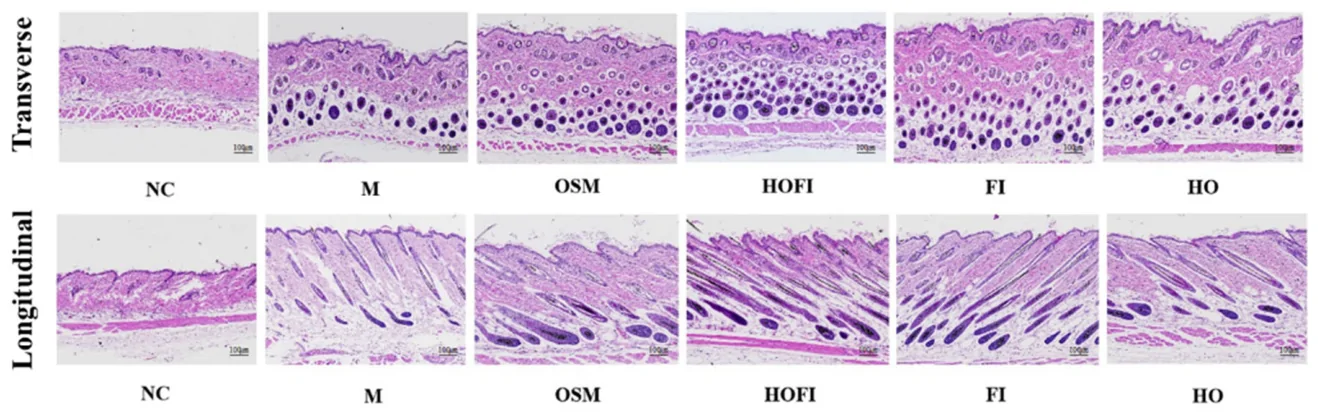
H&E staining of transverse and longitudinal skin tissue sections in physiological hair loss mice (scale: 100 μm).

After physiological hair loss, the hair cycle of mice went through three phases including anagen, telogen, and catagen phases [10]. During the anagen phase, the number, diameter, length, and melanin content of hair follicles gradually increased [27,28,29]. After twenty-one days of intervention, the results of skin tissue histological analysis are shown in Figure 5. The mice in the NC group were not depilated; their hair grew slowly, so the hair cycle of the mice was in the catagen phase. The results of skin tissue histological analysis in the NC group showed that the number of hair follicles was relatively few, the diameter of the follicles was small, and the cells of follicles were sparse. Compared with the NC group, the number, diameter, and melanin content of hair follicles in the M group were increased, confirming successful anagen induction by depilation. Based on transverse sections, compared with the M group, the number, diameter, and melanin content of hair follicles were increased in the OSM, HOFI, FI, and HO groups. Moreover, HOFI treatment resulted in the most significant increases in these parameters among all treatment groups. Based on longitudinal section, compared with the M group, the length of hair follicles was increased in the OSM, HOFI, FI, and HO groups. Moreover, the most pronounced increase in hair follicle length was observed in the HOFI group among all treatment groups. HOFI effectively promoted the longitudinal growth of hair follicles.

#### 3.2.4. Effects of HOFI on Dermal Thickness in Transverse and Longitudinal Sections in Physiological Alopecia Mice

**Figure 6:**
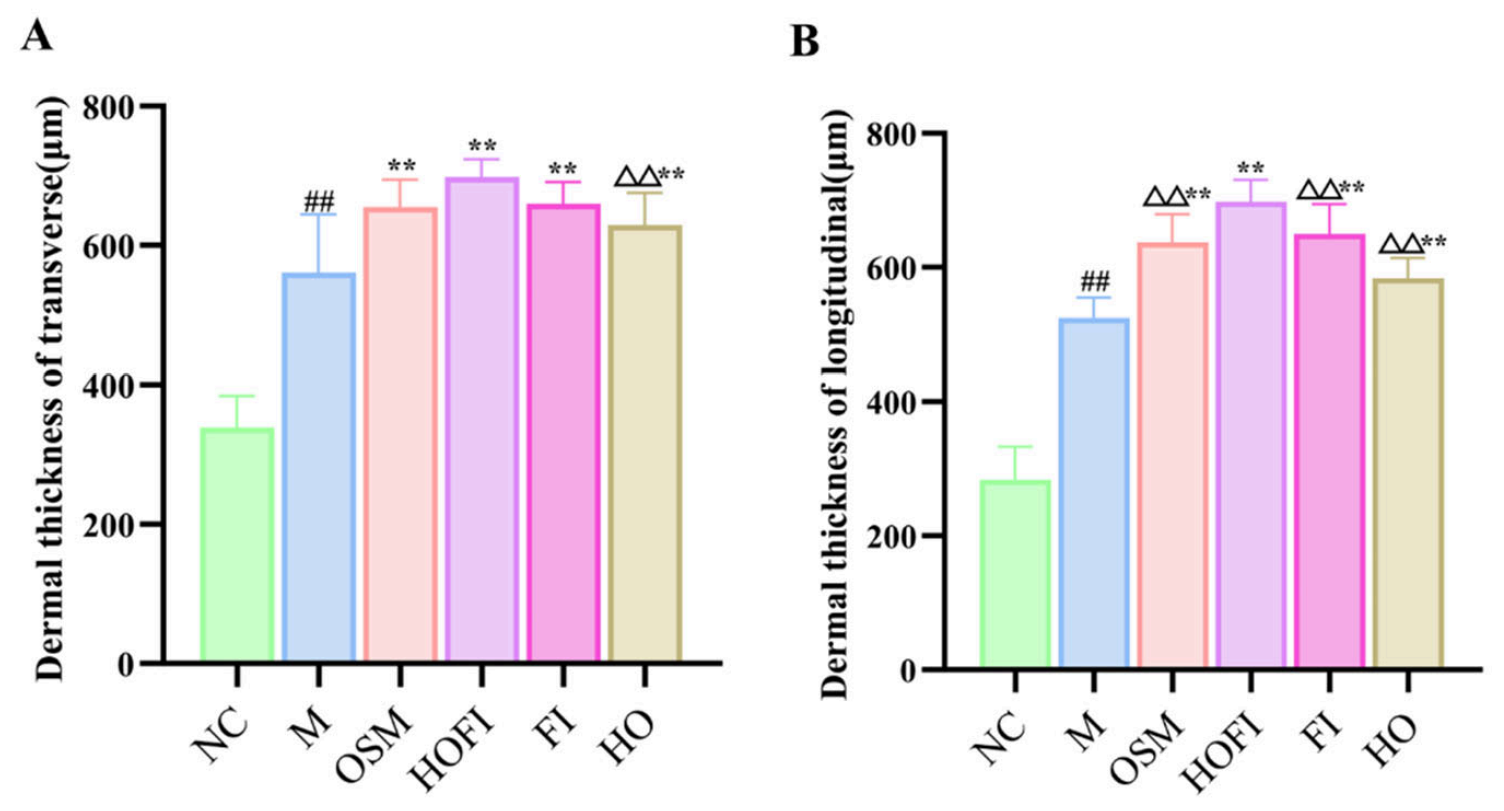
Dermal thickness of transverse and longitudinal skin tissue sections in physiological hair loss mice. (**A**) Dermal thickness of transverse skin tissue section; (**B**) dermal thickness of longitudinal skin tissue section. Data are presented as mean ± SD (*n* = 3). Annotations: *p* values denote the following comparisons: NC group (^##^ *p*  <  0.01), M group (** *p*  <  0.01), and HOFI group (^△△^ *p*  <  0.01).

Dermal thickness analysis of both transverse and longitudinal sections of skin tissue is regarded as a critical methodology for evaluating the growth phase of hair follicles [30,31]. During the anagen phase, hair follicle activity is significantly enhanced [32]. This phase is characterized by an increase in the number of hair follicles and expansion of the volume of individual follicles, accompanied by thickening of the dermis in the transverse section of the skin tissue [31]. The depth of hair follicle growth increased when follicles extended into the subcutaneous tissue, resulting in an increase in dermis thickness in the longitudinal section [33]. After twenty-one days of intervention, the histological analysis results of the skin tissue were shown in Figure 6. In the NC group, hair follicles remained arrested in the catagen phase, showing minimal proliferative activity and no spontaneous shift into anagen. On transverse sections, dermal thickness was significantly greater in the M group than in NC (*p*  <  0.01), further verifying successful anagen induction via depilation and confirming model establishment. Compared with M, the OSM, HOFI, FI, and HO groups all showed significantly increased dermal thickness (*p*  <  0.01). HOFI-treated samples were comparable to OSM and FI (*p*  >  0.05) but significantly thicker than HO (*p*  <  0.01). Similarly, on longitudinal sections, dermal thickness in OSM, HOFI, FI, and HO exceeded that in M (*p*  <  0.01), with HOFI reaching the highest value among treatment groups and significantly surpassing OSM, FI, and HO (*p*  <  0.01). Collectively, these findings indicate that HOFI promotes hair follicle activation and growth by driving the telogen-to-anagen transition, increasing follicle number, and enhancing follicular development along both sectional planes, ultimately thickening the dermis and highlighting its efficacy in stimulating hair growth.

#### 3.2.5. Effect of HOFI on the Level of VEGF in Physiological Alopecia Mice

**Figure 7:**
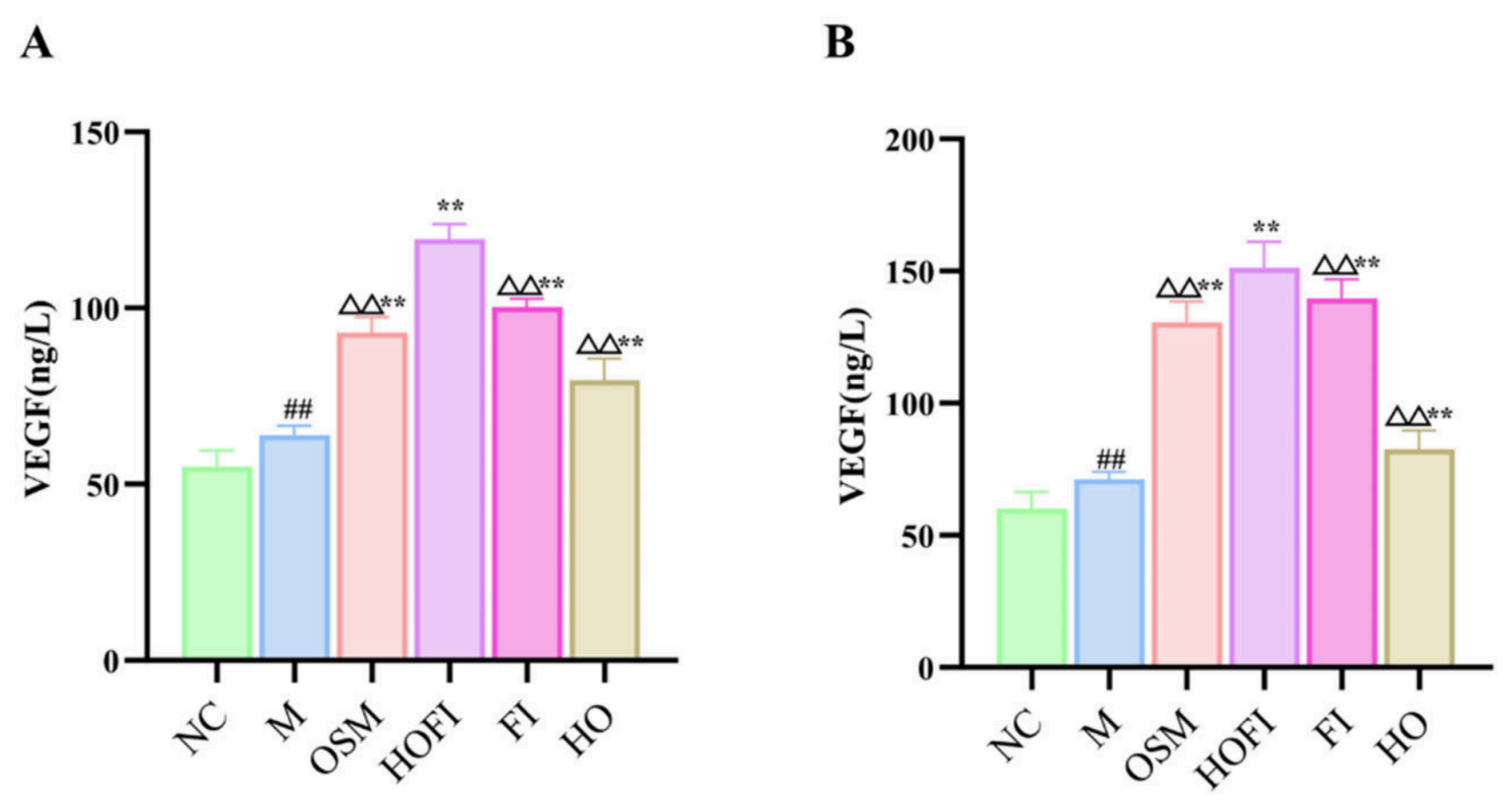
VEGF levels in plasma and skin tissue of physiological hair loss mice. (**A**) VEGF levels in plasma; (**B**) VEGF levels in skin tissue. Data are presented as mean ± SD (*n* = 3). Annotations: *p* values denote the following comparisons: NC group (^##^ *p*  <  0.01), M group (** *p*  <  0.01), and HOFI group (^△△^ *p*  <  0.01).

VEGF, a critical growth factor, promotes the proliferation, migration, and survival of endothelial cells [34]. VEGF is activated in response to hair follicle damage, and angiogenesis around the follicles is promoted, thereby ensuring an adequate supply of nutrients and oxygen to the follicles during the anagen phase and supporting hair growth and regeneration [35]. As shown in Figure 7A, the level of VEGF in plasma in the M group was substantially elevated compared to that in the NC group (*p* < 0.01), consistent with the expected VEGF upregulation during anagen-associated angiogenesis. The levels of VEGF in plasma in the OSM, HO, FI, and HOFI groups were markedly higher than those in the M group (*p* < 0.01). Notably, the HOFI group showed significantly higher plasma VEGF levels compared with each of the OSM, HO, and FI groups (*p* < 0.01). VEGF secretion is efficiently promoted by HOFI, which improves blood circulation around hair follicles, enhances nutrient delivery, and consequently promotes hair follicle regeneration and growth. Systemic metabolic and endocrine support for hair growth is reflected by the plasma VEGF contents, whereas the improvement of the local follicular microenvironment and specific regulatory mechanisms underlying hair growth are directly indicated by the VEGF contents [36]. As shown in Figure 7B, compared with the NC group, the level of VEGF in skin tissue in the M group was significantly higher (*p* < 0.01), consistent with the expected VEGF upregulation during anagen-associated angiogenesis. Compared with the M group, the levels of VEGF in skin tissue in the OSM, HOFI, FI, and HO groups increased (*p* < 0.01). Furthermore, HOFI induced the most pronounced increase in skin tissue VEGF levels among all treatment groups (*p* < 0.01). These results demonstrate that HOFI upregulates skin tissue VEGF level, thereby enhancing perifollicular blood circulation, which in turn promotes hair follicle growth.

#### 3.2.6. Effect of HOFI on the Level of TGF-β1 in Physiological Alopecia Mice

**Figure 8:**
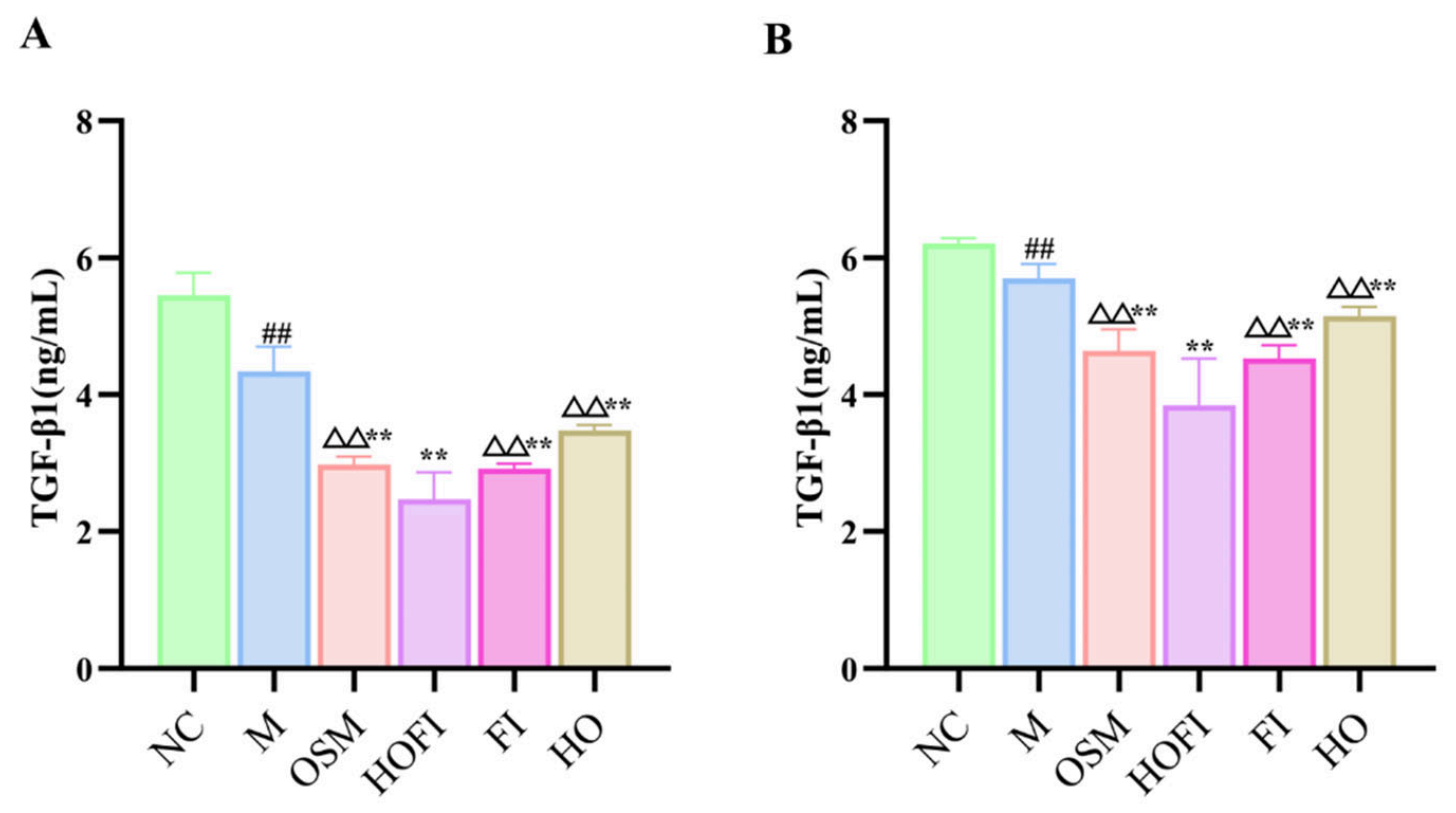
TGF-β1 levels in plasma and skin tissue of physiological hair loss mice. (**A**) TGF-β1 levels in plasma; (**B**) TGF-β1 levels in skin tissue. Data are presented as mean ± SD (*n* = 3). Annotations: *p* values denote the following comparisons: NC group (^##^ *p*  <  0.01), M group (** *p*  <  0.01), and HOFI group (^△△^ *p*  <  0.01).

The proliferation and differentiation of hair follicle stem cells are amplified by the inhibition of TGF-β1 secretion in hair follicles, leading to these stem cells being activated and sustained in an actively proliferative state, thereby prolonging the anagen phase and promoting hair growth [9]. Under normal physiological conditions, high levels of TGF-β1 expression are observed when hair follicles are in the telogen phase. Conversely, when hair follicles are damaged, they become activated to reduce TGF-β1 secretion [37]. As illustrated in Figure 8A, plasma TGF-β1 was markedly reduced in the M group relative to NC (*p*  <  0.01), reflecting the anticipated downregulation during anagen and confirming successful model establishment. Plasma TGF-β1 levels in the OSM, HO, FI, and HOFI groups were all significantly lower than in M (*p*  <  0.01). In particular, HOFI yielded the lowest plasma TGF-β1 among these cohorts, with values significantly below those of OSM, HO, and FI (*p*  <  0.01), implying stronger suppression of TGF-β1 expression and enhanced activation of hair follicle stem cells to support hair growth. Figure 8B shows a similar pattern in skin tissue: TGF-β1 was significantly lower in M than in NC (*p*  <  0.01), again consistent with anagen-associated downregulation and model validity. Compared with M, skin TGF-β1 decreased significantly in the OSM, HOFI, FI, and HO groups (*p*  <  0.01), with HOFI producing the most pronounced reduction among all treatments (*p*  <  0.01). Overall, HOFI effectively restrains TGF-β1 expression, alleviating its inhibitory effect on hair follicle stem cells and extending the anagen phase to promote hair growth.

#### 3.2.7. Effect of HOFI on the Modulation of the β-Catenin, Bcl-2 and Bax Signaling Pathway in Physiological Alopecia Mice

**Figure 9:**
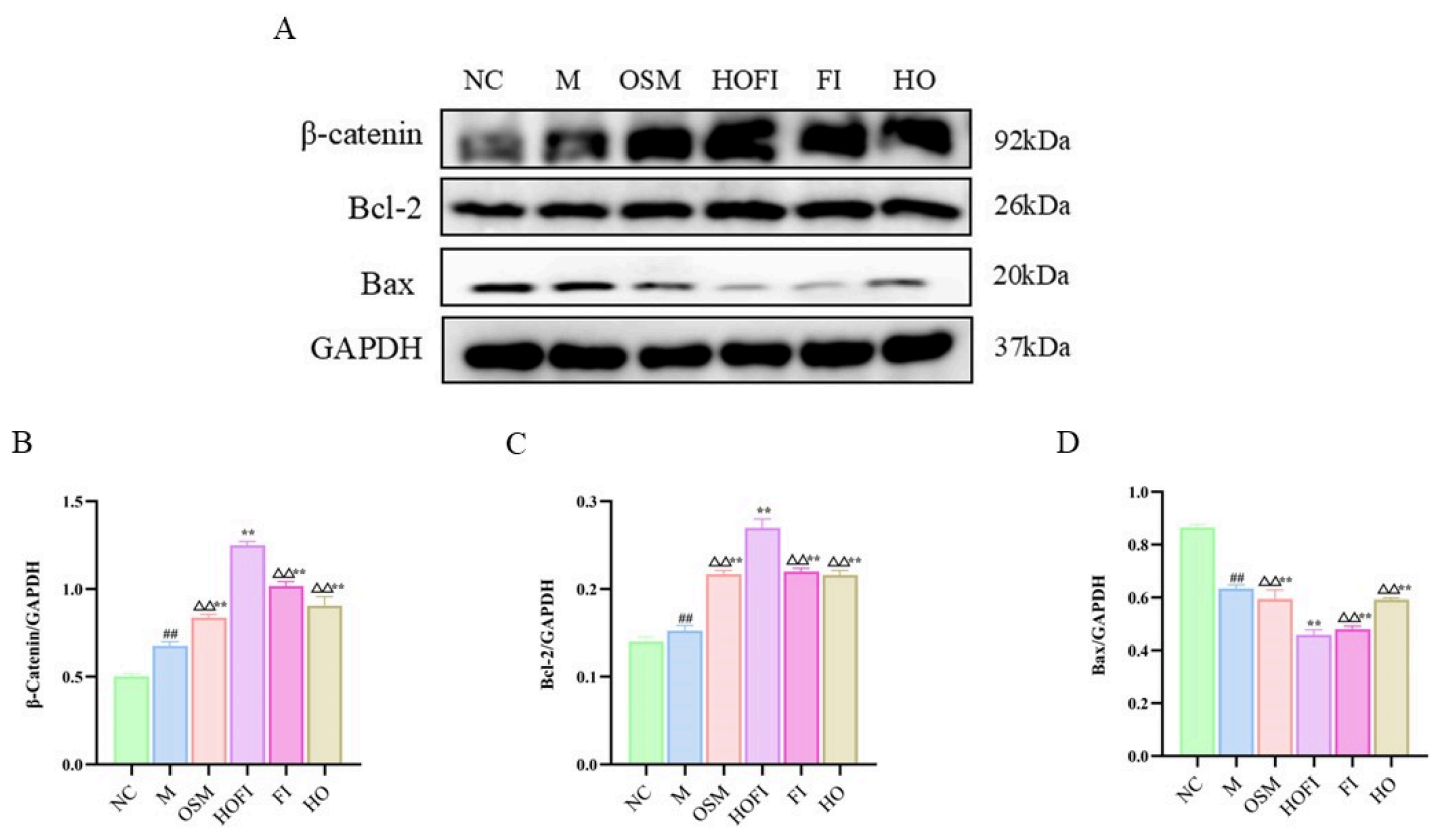
β-Catenin, Bcl-2 and Bax proteins expression levels in skin tissue of physiological hair loss mice. (**A**) The Western blot results for β-catenin, Bcl-2, Bax, and GAPDH proteins; (**B**) the β-catenin protein expression level; (**C**) the Bcl-2 protein expression level; (**D**) the Bax protein expression level. Data are presented as mean ± SD (*n* = 3). Annotations: *p* values denote the following comparisons: NC group (^##^ *p*  <  0.01), M group (** *p*  <  0.01), and HOFI group (^△△^ *p*  <  0.01).

The growth and development of hair follicles are significantly influenced by the β-catenin signaling pathway [38]. Reduction of β-catenin inhibits hair follicle cell proliferation, accelerates follicular regression, and results in hair loss [39]. Thus, β-catenin is recognized as a critical regulator of hair follicle growth and hair regeneration. Its dysregulation is implicated in impaired hair growth or hair loss. Under normal physiological conditions, low expression levels of β-catenin protein maintain the hair follicle in the telogen phase. Upon hair follicle injury, β-catenin secretion in the follicle is upregulated [40]. As shown in Figure 9B, compared with the NC group, the expression levels of β-catenin protein in the skin tissue of the M group were substantially increased (*p* < 0.01), further confirming successful anagen induction by depilation. Compared with the M group, the expression levels of β-catenin protein in the OSM, HOFI, FI, and HO groups were increased (*p* < 0.01). Furthermore, HOFI induced the most pronounced increase in β-catenin protein expression in skin tissue among all groups (*p* < 0.01). This suggests that HOFI activated the β-catenin signaling pathway, leading to the initiation of the hair follicle anagen phase and enhanced β-catenin protein expression, which consequently facilitated hair growth. The anti-apoptotic protein Bcl-2 and pro-apoptotic protein Bax are coordinately regulated to maintain the equilibrium of apoptosis, a fundamental biological process [37]. Therefore, the inhibition of follicular cell apoptosis via upregulation of Bcl-2 and downregulation of Bax contributes to the promotion of hair growth [41]. The upregulation of Bcl-2 and downregulation of Bax induced by hair follicle injury obstruct the transmission of apoptotic signals, thereby safeguarding cells from apoptosis [42]. As shown in Figure 9C, compared with the NC group, the expression level of Bcl-2 protein in the skin tissue of the M group was increased (*p* < 0.01), further confirming successful anagen induction by depilation. Compared with the M group, the expression levels of Bcl-2 protein in the OSM, HOFI, FI, and HO groups were increased (*p* < 0.01). Furthermore, HOFI induced the most pronounced increase in Bcl-2 protein expression in skin tissue among all groups (*p* < 0.01). This suggests that HOFI promotes hair growth by inhibiting follicular cell apoptosis and inducing apoptotic blockade. As shown in Figure 9D, compared with the NC group, the expression level of Bax protein in the skin tissue of the M group was decreased (*p* < 0.01), further confirming successful anagen induction by depilation. Compared with the M group, the expression levels of Bax protein in the OSM, HOFI, FI, and HO groups were decreased (*p* < 0.01). Furthermore, HOFI induced the most pronounced decrease in Bax protein expression in skin tissue among all groups (*p* < 0.01). This suggests that HOFI stimulates hair growth and regeneration by inhibiting the production of pro-apoptotic proteins.

### 3.3. Effects of HOFI on Promoting Hair Growth in Seborrheic Alopecia Mice

#### 3.3.1. Effects of HOFI on Hair Growth Situations in Seborrheic Alopecia Mice

**Figure 10:**
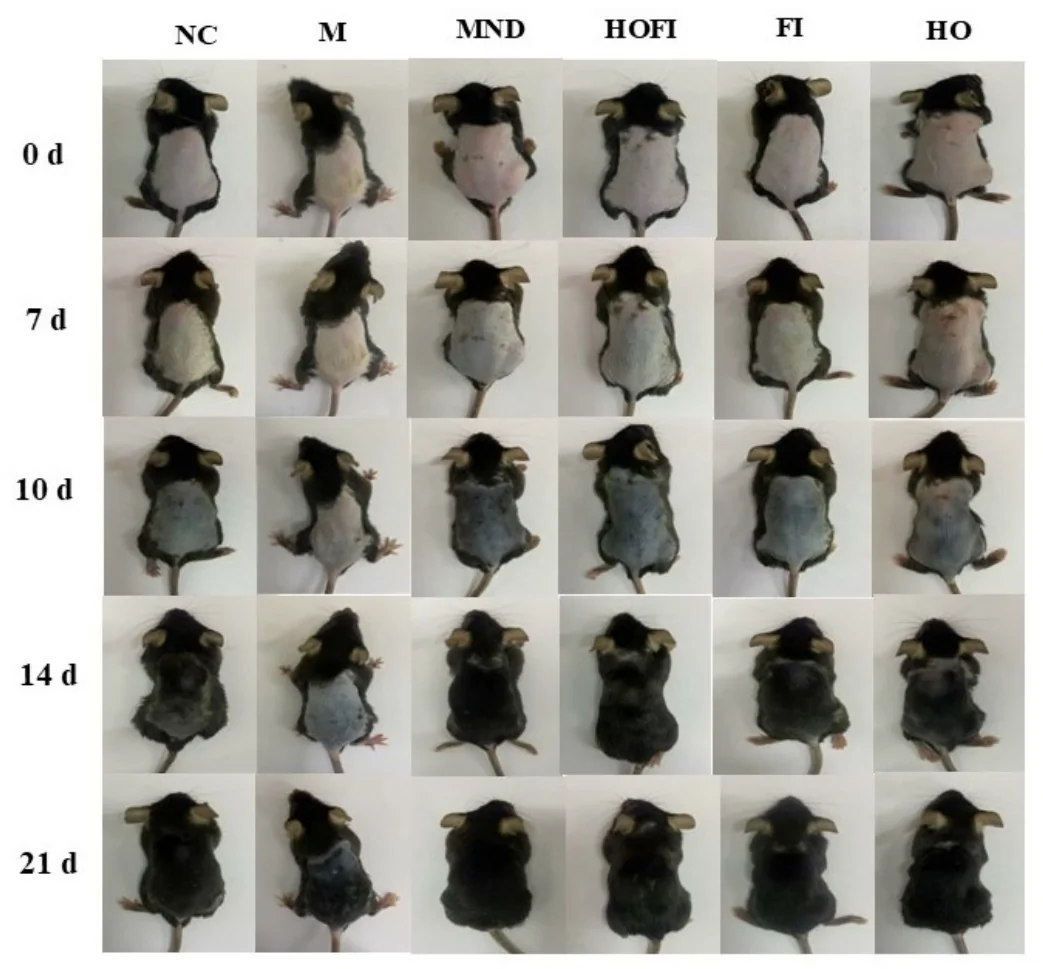
Effects of HOFI on hair growth situations in seborrheic hair loss mice.

As shown in Figure 10, following 5 weeks of subcutaneous testosterone administration (5 mg/kg/day), mice subjected to modeling (all groups except NC group) developed the characteristic phenotype of seborrheic alopecia, including sparse, dull, and brittle hair, reduced density, and oily skin. After depilation, the depilated dorsal region of the mice in these groups appeared pink, indicating the entry of hair follicles into the telogen phase. Collectively, these observations confirm the successful establishment of the mouse model of seborrheic alopecia. After seven days of intervention, the depilated dorsal region of the mice in the M group displayed light gray colors, indicating that sparse hair had grown. Compared with the M group, the depilated dorsal region of the mice in the HO, FI, HOFI, and MND groups displayed darker and darker colors, indicating more and more hair had grown. After interventions from the 7th to the 21st day, the depilated dorsal region of the mice in all groups displayed gradually darker colors, indicating that more hair had grown. Meanwhile, the depilated dorsal region of the mice in the MND, HOFI, FI, and HO groups displayed darker colors than those in the M group, indicating that there is more hair in the MND, HOFI, FI, and HO groups than that in the M group. Furthermore, the depilated dorsal region of the mice in the HOFI group exhibited a color comparable to that in the MND group, and both were darker than that in the FI and HO groups. The HOFI significantly promoted hair growth in mice with testosterone-induced seborrheic alopecia, exhibiting effects superior to those of individual FI or HO. This enhanced efficacy is likely attributable to a synergistic interaction between its components. Additionally, on the 7th day, the hair of the mice in each group had not yet grown, making it impossible to measure hair length. Based on the results of the preliminary experiment, 10 days was chosen as the starting point for measuring hair length.

#### 3.3.2. Effects of HOFI on Hair Length in Seborrheic Alopecia Mice

Table 3 shows that on intervention days 10, 14, and 21, hair length in the M group was significantly shorter than in the NC group (*p*  <  0.01), confirming successful establishment of the seborrheic alopecia mouse model. Over the same time points, hair length in the MND, HOFI, FI, and HO groups was significantly greater than in the M group (*p*  <  0.01). Notably, the MND group exhibited significantly longer hair than the HOFI group (*p*  <  0.01), while the HOFI group produced significantly longer hair than the FI and HO groups (*p*  <  0.01). Among all treatments, MND exhibited the most potent effect in promoting hair growth in seborrheic alopecia mice. Although the efficacy of HOFI was inferior to that of MND, it still surpassed that of HO or FI alone, indicating a synergistic or enhanced effect of the combined formulation.

#### 3.3.3. Regulation Effects of HOFI on Hair Follicles in Seborrheic Alopecia Mice

**Figure 11:**
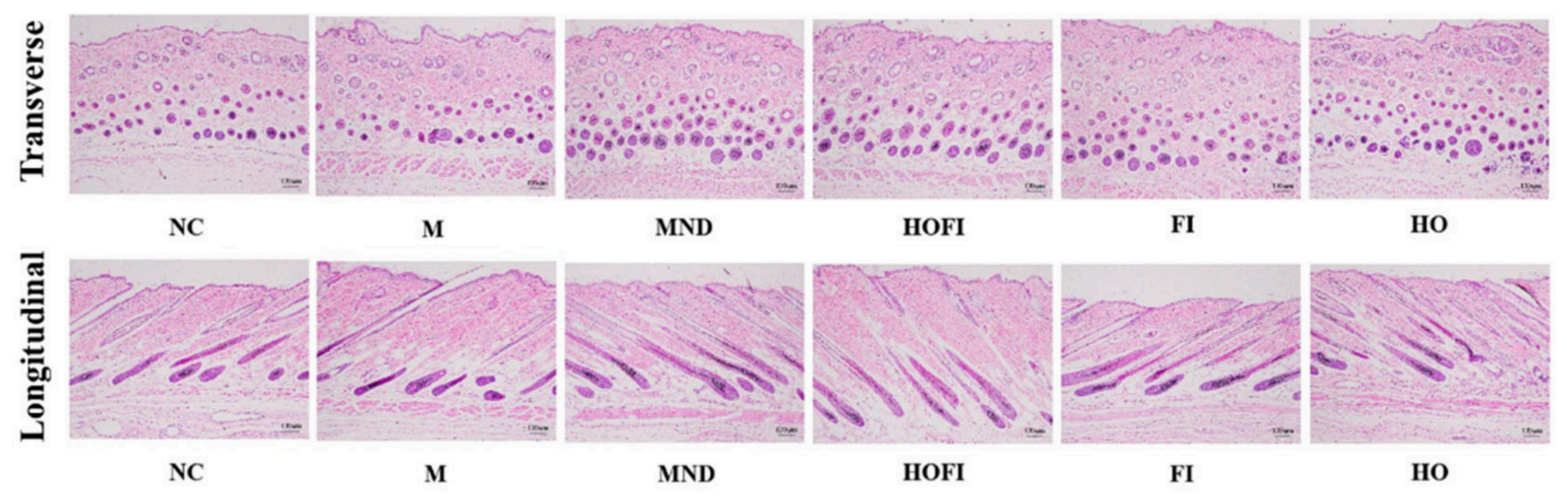
H&E staining of transverse and longitudinal skin tissue sections in seborrheic hair loss mice (scale: 100 μm).

Melanocytes are localized at the apex of the dermal papilla and are embedded within the hair shaft. These cells are active and produce melanin only during the anagen phase. Consequently, the presence of melanin within the follicle can be used as a reliable morphological indicator to confirm that the follicle is in the anagen phase [43]. After twenty-one days of intervention, the results of skin tissue histological analysis were shown in Figure 11. Based on transverse sections, compared with the NC group, hair follicles in the M group exhibited significant pathological alterations, including a marked reduction in follicular density and number, severe atrophy and deformation of morphology, and the presence of miniaturized follicles, collectively indicating successful model establishment. Compared with the M group, hair follicles in the OSM, HOFI, FI, and HO groups appeared increased in number, larger in diameter, and richer in melanin content. The HOFI group appeared comparable to the MND group but more pronounced than in the FI and HO groups. Based on longitudinal section, compared with the NC group, hair follicles in the M group exhibited significant pathological alterations, including severe follicular atrophy, a marked reduction in follicle depth, and disorganized cellular arrangement within the follicles, collectively indicating successful model establishment. Compared with the M group, hair follicles in the OSM, HOFI, FI, and HO groups exhibited increased length, with characteristics of deep dermal extension, a thick hair shaft, and tight tissue integration. The HOFI group appeared comparable to those in the MND group but more pronounced than in the FI and HO groups. This suggests that HOFI effectively promoted the longitudinal growth of hair follicles, thereby maintaining their normal physiological architecture.

#### 3.3.4. Effects of HOFI on Dermal Thickness in Transverse and Longitudinal Sections in Seborrheic Alopecia Mice

**Figure 12:**
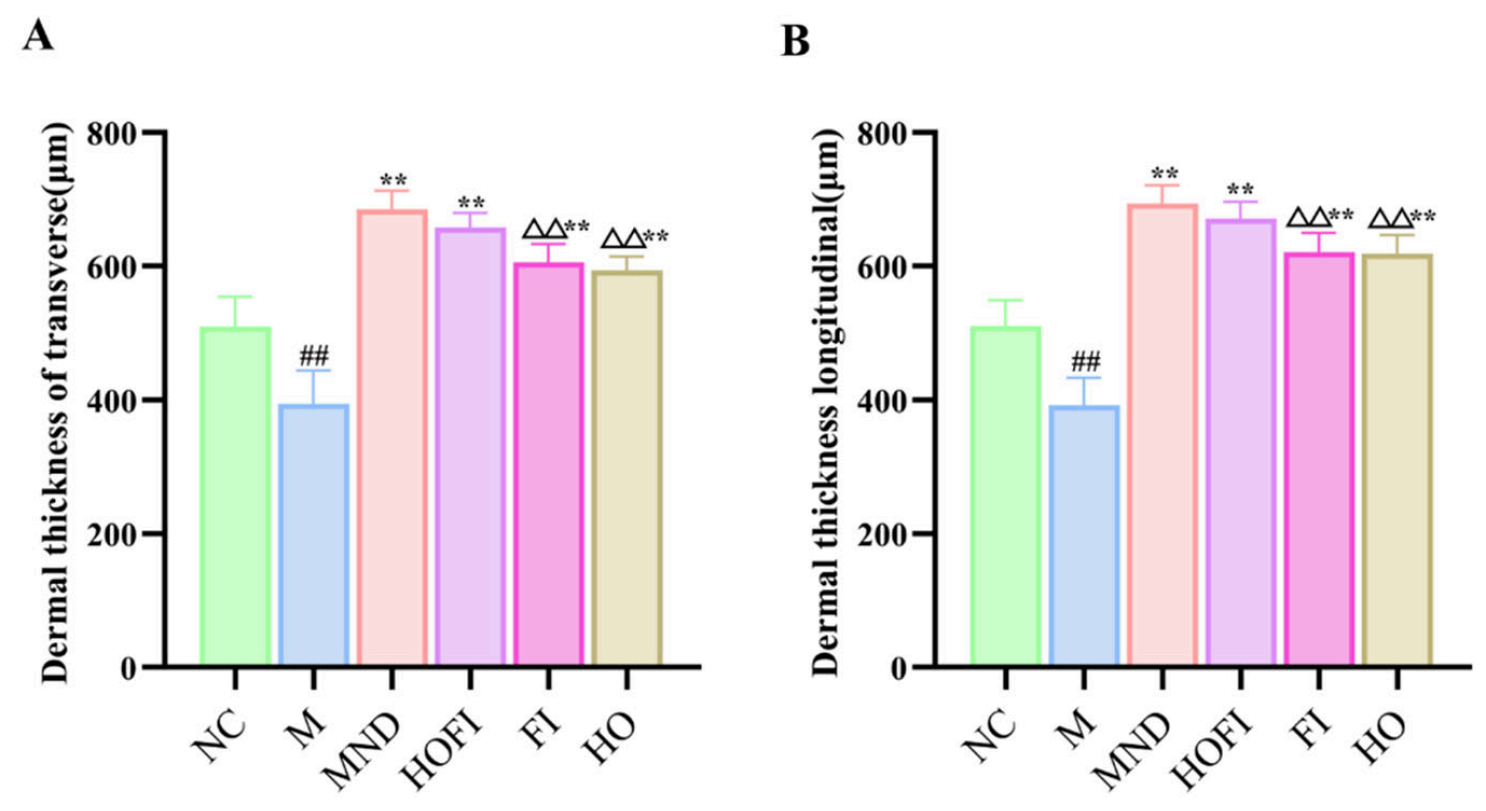
Dermal thickness of transverse and longitudinal skin tissue sections in seborrheic hair loss mice dorsal skin. (**A**) Dermal thickness of transverse skin tissue section; (**B**) dermal thickness of longitudinal skin tissue section. Data are presented as mean ± SD (*n* = 3). Annotations: *p* values denote the following comparisons: NC group (^##^ *p*  <  0.01), M group (** *p*  <  0.01), and HOFI group (^△△^ *p*  <  0.01).

As illustrated in Figure 12, transverse and longitudinal sections revealed markedly reduced dermal thickness in the M group relative to NC (*p*  <  0.01), confirming successful model induction. Dermal thickness was significantly increased in the OSM, HOFI, FI, and HO groups compared with M (*p*  <  0.01). The HOFI group showed dermal thickness similar to MND yet significantly greater than FI and HO (*p*  <  0.01). These findings indicate that HOFI facilitates the shift of hair follicles from telogen to anagen, leading to a higher follicle count, improved structural morphology, and enhanced follicular development. Ultimately, this contributes to increased dermal thickness and underscores the pronounced efficacy of HOFI in promoting hair growth.

#### 3.3.5. Effect of HOFI on VEGF Levels in Seborrheic Alopecia Mice

**Figure 13:**
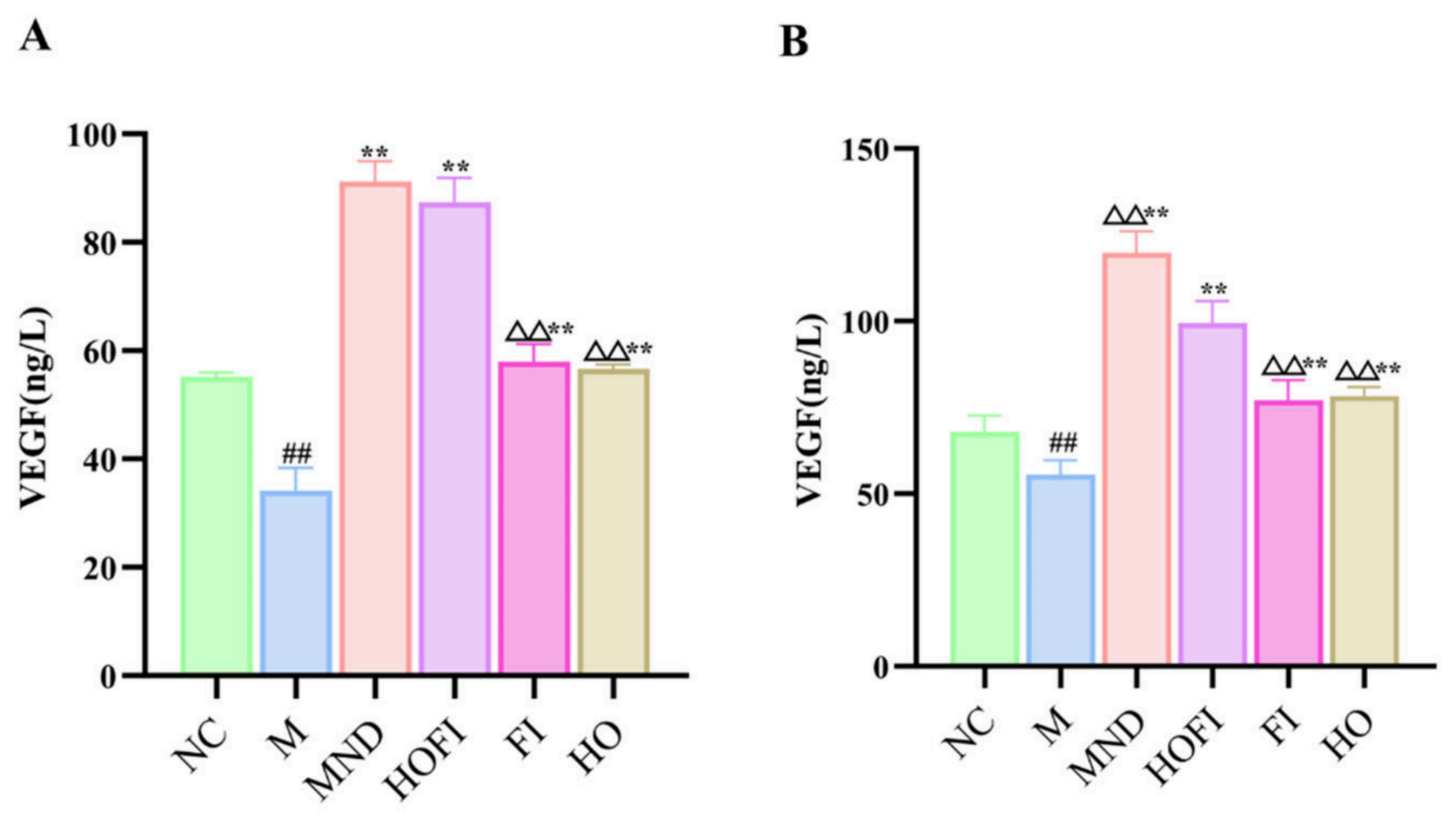
VEGF levels in plasma and skin tissue of seborrheic hair loss mice. (**A**) VEGF levels in plasma; (**B**) VEGF levels in skin tissue. Data are presented as mean ± SD (*n* = 3). Annotations: *p* values denote the following comparisons: NC group (^##^ *p*  <  0.01), M group (** *p*  <  0.01), and HOFI group (^△△^ *p*  <  0.01).

Figure 13A shows that plasma VEGF levels were significantly lower in the M group than in NC (*p*  <  0.01), confirming successful model establishment. Relative to M, VEGF was significantly elevated in the MND, HOFI, FI, and HO groups (*p*  <  0.01). HOFI achieved VEGF levels comparable to MND, yet significantly lower than FI and HO (*p*  <  0.01). Minoxidil is known to improve perifollicular circulation and stimulate VEGF secretion, thereby helping hair follicles enter the anagen phase and promoting hair growth. [44]. Its mechanism includes opening potassium channels in dermal papilla cells and upregulating VEGF secretion, thereby improving blood supply and nutrient delivery to hair follicles [45,46]. These findings suggest that HOFI elevates plasma VEGF by opening potassium channels in dermal papilla cells and boosting VEGF secretion. Figure 13B shows that plasma VEGF was significantly reduced in the M group relative to NC (*p*  <  0.01), confirming successful model establishment. Compared with M, VEGF increased significantly in the MND, HOFI, FI, and HO groups (*p*  <  0.01). HOFI produced significantly lower VEGF levels than MND (*p*  <  0.01), but notably higher levels than FI and HO (*p*  <  0.01). Collectively, these results indicate that HOFI enhances epidermal VEGF secretion and improves perifollicular microcirculation, thereby supporting hair follicle regeneration and growth.

#### 3.3.6. Effect of HOFI on TGF-β1 Levels in Seborrheic Alopecia Mice

**Figure 14:**
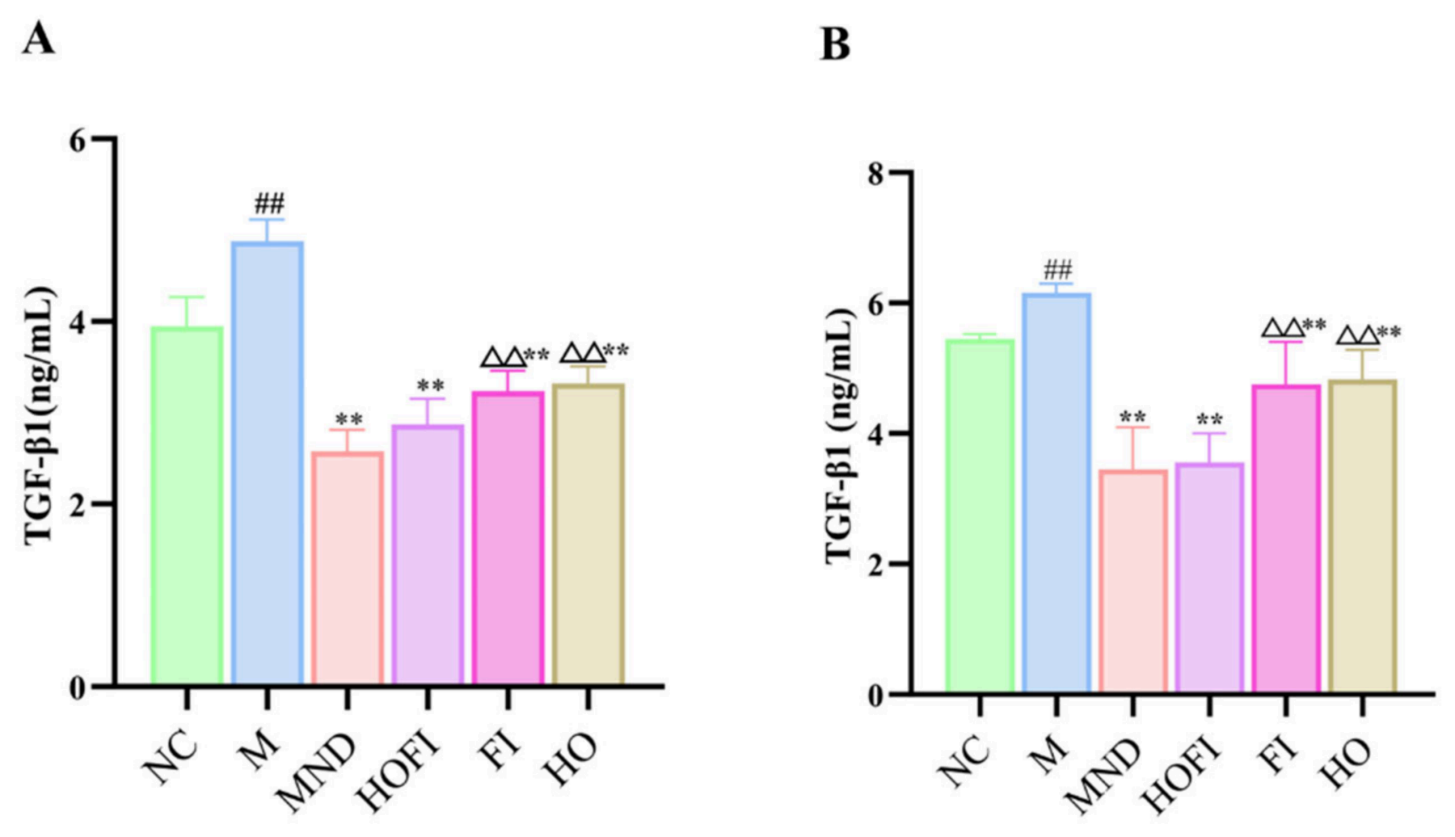
TGF-β1 levels in plasma and skin tissue of seborrheic hair loss mice. (**A**) TGF-β1 levels in plasma; (**B**) TGF-β1 levels in skin tissue. Data are presented as mean ± SD (*n* = 3). Annotations: *p* values denote the following comparisons: NC group (^##^ *p*  <  0.01), M group (** *p*  <  0.01), and HOFI group (^△△^ *p*  <  0.01).

Seborrheic alopecia may be associated with elevated TGF-β1, which induces apoptosis in hair follicle epithelial cells and hastens the shift of follicles from telogen to catagen, thereby contributing to hair loss [26]. Accordingly, lowering TGF-β1 represents a promising therapeutic approach for alopecia [47]. Figure 14A shows that plasma TGF-β1 was significantly higher in the M group than in NC (*p*  <  0.01), confirming model success. Relative to M, TGF-β1 decreased significantly in the MND, HOFI, FI, and HO groups (*p*  <  0.01). HOFI produced levels similar to MND, yet markedly lower than those in FI and HO (*p*  <  0.01).

Figure 14B shows that skin tissue TGF-β1 was significantly elevated in the M group relative to NC (*p*  <  0.01), confirming successful model establishment. Versus M, TGF-β1 levels were significantly reduced in the MND, HOFI, FI, and HO groups (*p*  <  0.01). HOFI yielded values similar to MND yet markedly lower than FI and HO (*p*  <  0.01). These results indicate that HOFI exerts a synergistic effect by suppressing TGF-β1 release and promoting hair follicle stem cell activation, thus supporting hair regeneration and growth.

#### 3.3.7. Effect of HOFI on the Modulation of the β-Catenin, Bcl-2 and Bax Signaling Pathway in Seborrheic Alopecia Mice

**Figure 15:**
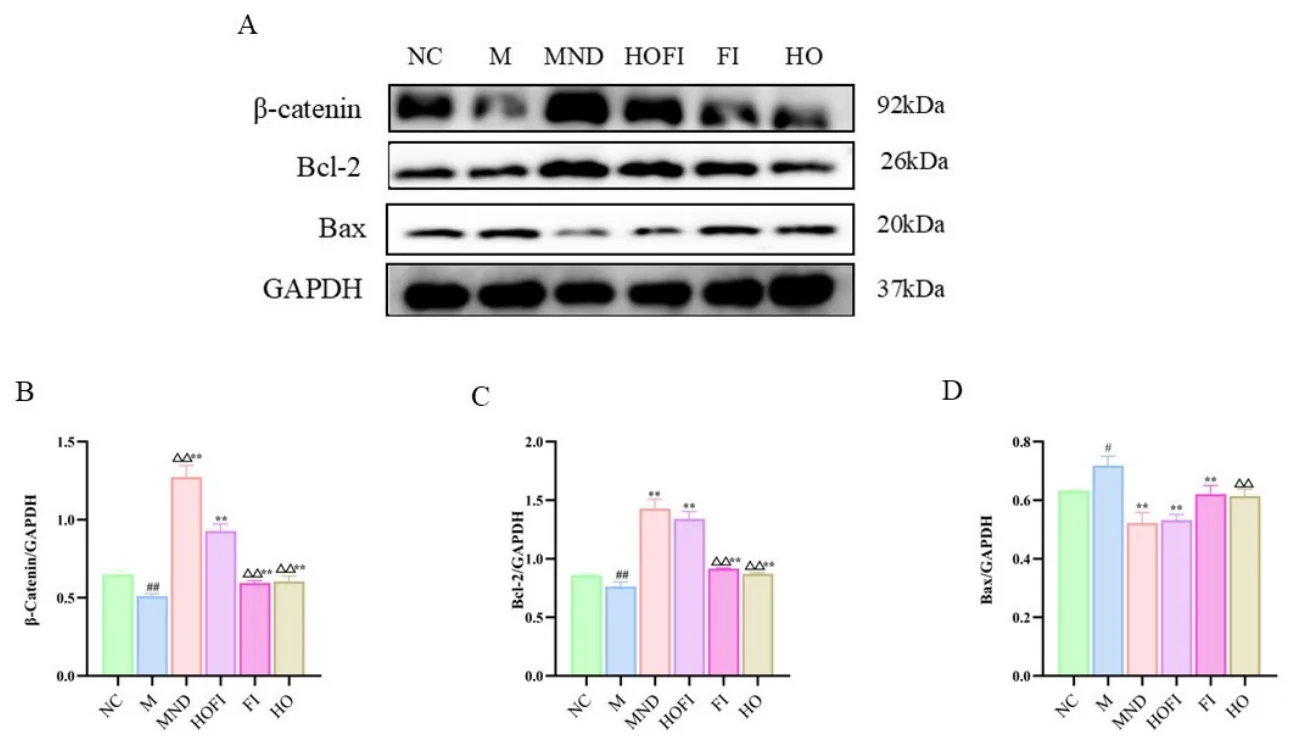
(**A**) Western blot bands for β-catenin, Bcl-2, Bax, and GAPDH; (**B**–**D**) Quantified protein levels of β-catenin, Bcl-2, and Bax, respectively. Values are expressed as mean ± SD (*n* = 3). Annotations: *p* values denote the following comparisons: NC group (^#^ *p*  <  0.05, ^##^ *p*  <  0.01), M group (** *p*  <  0.01), and HOFI group (^△△^ *p*  <  0.01).

As illustrated in Figure 15B, skin tissue β-catenin levels were significantly reduced in the M group relative to the NC group (*p*  <  0.01), indicating that the model was successfully established. Compared with the M group, β-catenin expression in the MND, HOFI, FI, and HO groups was increased (*p* < 0.01). β-catenin expression in the HOFI group was significantly lower than in the MND group (*p*  <  0.01), but notably higher than in the FI and HO groups (*p*  <  0.01). These findings imply that HOFI triggers the β-catenin signaling cascade, thereby promoting entry into the hair follicle anagen phase and upregulating β-catenin protein levels, ultimately supporting hair growth. Figure 15C shows that Bcl-2 protein levels in skin tissue were significantly lower in the M group than in the NC group (*p*  <  0.01), confirming successful model establishment. Relative to the M group, Bcl-2 expression was elevated in the MND, HOFI, FI, and HO groups (*p*  <  0.01). HOFI produced Bcl-2 levels similar to those in the MND group, but markedly greater than in the FI and HO groups (*p*  <  0.01). These results imply that HOFI supports hair growth by suppressing follicular cell apoptosis and blocking the apoptotic process. Figure 15D shows that Bax protein levels in skin tissue were significantly elevated in the M group relative to NC (*p*  <  0.05), confirming successful model establishment. Compared with M, Bax expression decreased in the MND, HOFI, and FI groups (*p*  <  0.01). HOFI yielded Bax levels similar to those in MND and FI, yet markedly lower than in HO (*p*  <  0.01). These findings imply that HOFI promotes hair growth and regeneration by suppressing pro-apoptotic protein production.

## 4. Discussion

HO contains abundant unsaturated fatty acids, such as linoleic and arachidonic acids, which have been shown to promote hair growth [20,21]. In particular, linoleic acid stimulates dermal papilla cell proliferation through β-catenin pathway activation, while arachidonic acid has been reported to promote hair growth in a seborrheic alopecia mouse model [1,21]. FI contains active compounds such as indigotin and indirubin [48]. This finding is consistent with a previous C57BL/6 study in which co-administration of Laminaria japonica and Cistanche tubulosa extracts promoted hair regrowth more effectively than either single extract alone, supporting the rationale for multi-component herbal formulations [49].

Hair follicles undergo transitions through distinct phases: anagen phase, catagen phase, and telogen phase [50]. Depilation induces hair follicles into a new anagen phase [51]. In this study, HOFI significantly accelerated the depilated dorsal region darkening process and increased hair length, indicating its ability to enter the anagen phase prematurely. Histological examination showed that HOFI markedly increased hair follicle number and size and enhanced dermal thickness. HOFI stimulated hair follicles to exit telogen and enter the active growth cycle. These findings are consistent with previous C57BL/6 depilation studies using low-molecular-weight fish collagen peptide [52] and centipedegrass extract [53], which similarly reported skin darkening, increased hair length, follicular enlargement and dermal thickening as indicators of premature telogen-to-anagen transition.

The β-catenin pathway serves as the master regulator for anagen initiation and follicular stem cell activation [54]. The β-catenin pathway is a well-established mechanism by which various phytochemicals promote hair growth [55]. HOFI markedly enhanced β-catenin expression in skin tissue, which mechanistically accounts for its ability to promote the telogen-to-anagen shift and elevate hair follicle density. Consistent with this, *P. mira* nut oil facilitates hair growth through regulation of the β-catenin signaling axis [56]. Notably, other botanical oils evaluated in C57BL/6 models additionally activated the Shh/Gli signaling cascade alongside Wnt/β-catenin [9], suggesting that the pro-hair-growth mechanism of HOFI may extend beyond the targets examined here. VEGF is a potent mitogen and angiogenic factor whose secretion by dermal papilla cells is essential for sustaining anagen [57]. The results from Panax japonicus and other herbs studied here suggest that the traditional Chinese medicine formulation promotes hair growth by enhancing VEGF expression [34]. Conversely, TGF-β1 is a potent negative regulator that induces follicle regression by inhibiting keratinocyte proliferation and promoting apoptosis; its suppression is a recognized therapeutic strategy [31]. HOFI not only elevated VEGF levels but also suppressed TGF-β1 in both plasma and skin tissue. Notably, the Centipeda minima extract DN106212 in the same C57BL/6 model concurrently up-regulated VEGF and down-regulated TGF-β1 while additionally raising IGF-1 [58], suggesting that the VEGF–TGF-β1 modulation by HOFI may operate alongside other growth factor axes not examined here. A high Bcl-2/Bax ratio is a hallmark of cell survival during the anagen phase, protecting follicles from premature entry into catagen [59]. The extract of Salvia plebeia exerted anti-apoptotic and proliferative effects by increasing the Bcl-2/Bax ratio [60]. Arachidonic acid has similarly been reported to prolong the anagen phase in C57BL/6 mice and to increase Bcl-2 expression in dermal papilla cells via ERK/AKT phosphorylation [21], supporting our finding that the HOFI group showed reduced Bax and elevated Bcl-2. HOFI increased Bcl-2 and decreased Bax expression indicates a potent anti-apoptotic activity at the cellular level, which synergizes with the pro-growth signals from the Wnt and VEGF pathways to maintain follicular integrity. These mechanisms provide a comprehensive molecular explanation for its efficacy in promoting hair growth in both the telogen-to-anagen transition model and the seborrheic alopecia model. This study has several limitations that should be noted. First, the protein panel was limited to five preselected targets; whether additional Wnt-related mediators (e.g., Wnt10b, Lef1, Axin2) or parallel pathways (e.g., Shh, Notch, PI3K/Akt) are involved remains to be elucidated. Second, the seborrheic alopecia model was induced by exogenous testosterone, which reproduces androgen-driven miniaturization but does not capture the chronic inflammatory microenvironment or genetic predisposition characteristic of human androgenetic alopecia. Third, the 21-day observation period covered the telogen-to-anagen transition and early anagen maintenance, whereas the long-term persistence of newly formed hairs after treatment cessation was not examined. Finally, while the two mouse models employed here represent distinct alopecia subtypes, extrapolation to human patients requires caution given the substantial differences in hair cycle duration, follicle density, and immune microenvironment between rodent skin and human scalp. Additionally, the HO and FI used in this study were crude extracts, which may exhibit batch-to-batch variation in composition. Future studies should focus on standardization of the extract by quantifying marker compounds and/or normalizing biological activity to defined phytochemical content. This will enhance reproducibility, allow meaningful comparison across studies, and strengthen the translational value of the findings. It should be noted that the specificity of the antibodies used in this study relies on manufacturer validation rather than in-house knockout or blocking-peptide controls, which represents a limitation of the present work.

## 5. Conclusions

To sum up, the HO, FI and HOFI can significantly increase hair length, improve hair follicle number and size, and thicken the dermis, thereby significantly promoting hair growth. Notably, HOFI treatment induced a markedly stronger hair growth-promoting effect compared with the FI and HO treatments. The underlying mechanisms of their ability to promote hair growth are related with increasing VEGF levels, decreasing TGF-β1 levels, and activating the Wnt/β-catenin pathway. Meanwhile, HOFI can upregulate Bcl-2 and suppress Bax expression, thereby inhibiting the apoptosis of hair follicle cells in skin tissue. These findings provide strong preliminary evidence that the HOFI topical preparation possesses hair growth-promoting properties. Standardized preparations and further validation will be essential to confirm reproducibility and translational relevance.

## Figures and Tables

**Table 1:** Determination results of sample content (X ± SD, n = 3).

No.	Eyebrow Pencil Batch	Indigo (µg/g)	Indirubin (µg/g)
1	2024.03.06	1174.638 ± 32.521	79.441 ± 2.668
2	2024.04.02 a	1151.367 ± 20.339	79.274 ± 2.769
3	2024.04.02 b	1105.144 ± 28.441	74.905 ± 3.934
4	2024.04.02 c	1135.42 ± 4.332	77.797 ± 1.86
5	2024.05.05	1140.373 ± 39.726	77.553 ± 1.573
6	2024.05.06	1099.423 ± 91.446	75.072 ± 3.528
7	2024.06.15 a	1127.070 ± 55.964	76.687 ± 1.968
8	2024.06.15 b	1194.58 ± 157.802	81.628 ± 9.024
9	2024.06.30	1139.535 ± 45.735	75.652 ± 3.826
10	2024.07.05 a	1134.394 ± 52.774	76.525 ± 3.673
11	2024.07.05 b	1104.973 ± 30.950	73.540 ± 1.775
12	2024.07.05 c	1141.278 ± 68.510	76.527 ± 5.356
13	2024.07.06 a	1153.038 ± 94.474	77.133 ± 6.902
14	2024.07.06 b	1145.488 ± 36.722	81.309 ± 1.666
15	2024.07.06 c	1137.819 ± 37.176	79.558 ± 0.922

Note: Different letters (a, b, c, etc.) denote independent batches prepared daily. The number of batches per day varies (two or three batches).

**Table 2:** Effect of HOFI on hair length in physiological hair loss mice (X ± SD, *n* = 10).

Groups	Hair Length (mm)		
	Day 10	Day 14	Day 21
NC	8.18 ± 0.48	8.21 ± 0.45	8.10 ± 0.43
M	1.10 ± 0.34 ^##^	3.11 ± 0.70 ^##^	5.25 ± 0.87 ^##^
OSM	2.46 ± 0.44 **^△△^	3.46 ± 0.87 **^△△^	6.15 ± 1.10 **^△△^
HOFI	2.68 ± 0.56 **	3.72 ± 0.99 **	6.39 ± 0.96 **
FI	2.40 ± 0.55 **^△△^	3.53 ± 0.96 **^△△^	6.05 ± 0.82 **^△△^
HO	1.17 ± 0.34 **^△△^	3.37 ± 0.79 **^△△^	5.45 ± 0.54 **^△△^

Annotations: *p* values denote the following comparisons: NC group (^##^ *p*  <  0.01), M group (** *p*  <  0.01), and HOFI group (^△△^ *p*  <  0.01).

**Table 3:** Effect of HOFI on hair length in seborrheic hair loss mice (X ± SD, n = 10).

Groups	Hair Length (mm)		
	Day 10	Day 14	Day 21
NC	1.10 ± 0.32	2.94 ± 0.42	5.02 ± 0.74
M	0.85 ± 0.21 ^##^	2.63 ± 0.41 ^##^	4.53 ± 0.78 ^##^
MND	2.34 ± 0.48 **^△△^	3.75 ± 1.11 **^△△^	5.63 ± 0.81 **^△△^
HOFI	2.25 ± 0.54 **	3.59 ± 1.07 **	5.50 ± 0.68 **
FI	1.95 ± 0.51 **^△△^	3.30 ± 0.83 **^△△^	5.16 ± 0.93 **^△△^
HO	1.90 ± 0.40 **^△△^	3.26 ± 0.83 **^△△^	5.16 ± 0.83 **^△△^

Annotations: *p* values denote the following comparisons: NC group (^##^ *p*  <  0.01), M group (** *p*  <  0.01), and HOFI group (^△△^ *p*  <  0.01).

## Data Availability

The original contributions presented in this study are included in the article. Further inquiries can be directed to the corresponding authors.

## Abbreviations

The following abbreviations are used in this manuscript:

Bcl-2	B-cell lymphoma 2
β-catenin	Beta-catenin
BCA	Bicinchoninic Acid Assay
Bax	Bcl-2-associated X protein
FI	Folium Isatidis Eyebrow Pencil
GAPDH	Glyceraldehyde-3-Phosphate Dehydrogenase
HE	Hematoxylin and Eosin Staining
HOFI	Horse Oil Folium Isatidis Eyebrow Pencil
HO	Horse Oil Eyebrow Pencil
MND	Minoxidil
M	Model
NC	Normal Control
OSM	Osman Eyebrow Growth Pencil
PBS	Phosphate-Buffered Saline
PVDF	Polyvinylidene Fluoride membrane
RSD	Relative Standard Deviation
TBST	Tris-Buffered Saline with Tween
TGF-β1	Transforming Growth Factor-β1
VEGF	Vascular Endothelial Growth Factor
Wnt/β-catenin	Wingless-type MMTV integration site family member 1/beta-catenin signaling pathway
